# Deep dermal and subcutaneous canine hemangiosarcoma in the perianal area: diagnosis of perianal mass in a dog

**DOI:** 10.1186/s12917-019-1852-6

**Published:** 2019-04-15

**Authors:** Eun Wha Choi

**Affiliations:** Department of Veterinary Clinical Pathology, College of Veterinary Medicine & Institute of Veterinary Science, Kangwon National University, 1 Kangwondaehak-gil, Chuncheon, Gangwon-do 24341 Republic of Korea

**Keywords:** Hemangiosarcoma, Perianal mass, Dog, Diagnostic cytology, CD31, Factor VIII-related antigen

## Abstract

**Background:**

Tumors of the perianal area occur frequently in dogs, and the two most common tumors are perianal gland adenoma and anal sac adenocarcinoma; others such as mast cell tumor, lymphoma and melanoma can also occur at this site. Diagnostic cytology is a useful technique and is usually used to establish a definitive diagnosis of some tumors in veterinary medicine. This report describes an extremely rare case of a deep dermal and subcutaneous canine hemangiosarcoma in the perianal area.

**Case presentation:**

A 13-year-old intact male spaniel was presented for evaluation of a 4 × 4 cm, ulcerated, and hemorrhagic mass presented in the right perianal region. In cytologic evaluation, malignant mesenchymal tumor with inflammation was diagnosed, and incidental heart worm microfilaremia was identified. Based on the cytologic evaluation, a punch biopsy (3 mm, three sites) was conducted under anesthesia and deep dermal and subcutaneous hemangiosarcoma (3 mitotic figures/10 high power field (400×)) was diagnosed by histopathological evaluation. It was also confirmed by immunohistochemistry results for cluster of differentiation 31 (CD31) and factor VIII-related antigen marker.

**Conclusions:**

Deep dermal and subcutaneous hemangiosarcoma in the perianal region is a rare condition, and its prognosis is usually poor. Perianal gland adenoma and anal sac adenocarcinoma are the two most common tumors in the perianal region, but other different types of tumors may also occur as in this case; therefore, accurate diagnosis is required using cytology and/or histopathological examination.

## Background

Tumors of the perianal area occur frequently in dogs, and the two most common tumors are perianal gland adenoma and anal sac adenocarcinoma [1]. Diagnostic cytology is a useful technique and is usually used to establish the diagnosis of these tumors [2]. Benign adenomas constitute over 81% of tumors of the perianal glands. Perianal gland adenoma is common in older intact male dogs, but can occur in younger, neutered, or female dogs. Treatment comprises tumor removal concomitant with orchiectomy; the prognosis is good and recurrence rates are less than 10% [1]. Sheets and clusters of large ovoid to cuboidal cells with round nuclei and abundant cytoplasm, similar to hepatocytes, are observed in aspirates from this tumor. Thus, it is also called “hepatoid tumor” [2]. Anal sac adenocarcinoma mostly occurs in older dogs, and about one-third of the affected dogs are presented with difficulty in defecation. Dogs with and those without pulmonary metastasis survived a median of 219 and 548 days, respectively; hypercalcemic dogs and dogs with tumors larger than 10 cm^2^ had a short survival time, with a median of 256 and 292 days, respectively [3]. Aspirates from anal sac adenocarcinoma show variably sized sheets of cells and cells with round nuclei, a moderate amount of pale cytoplasm, and indistinct cytoplasmic borders [2]. Cells from perianal gland adenoma and anal sac adenocarcinoma are of epithelial origin; thus, cellularity in the slides is usually high.

Cellularity in cytologic samples of tumors of mesenchymal origin is usually low but may be moderate to high. Tumors of mesenchymal origin are characterized by spindle-shaped cells and abundant intercellular eosinophilic matrix may be observed [2].

In tumors of mesenchymal origin, the origin of the constituent cells is difficult to determine by diagnostic cytology [2].

Hemangiosarcoma is a malignant mesenchymal tumor arising from vascular endothelial cells that can develop in any tissue, but the most frequent primary locations in dogs are the spleen (28–63%), right atrium and auricle (3–50%) [4]. Prognosis of visceral hemangiosarcoma is poor because of metastasis through hematologic routes or local seeding after tumor rupture [5]. Cytologic diagnosis of hemangiosarcoma can be challenging for cytopathologists, and histopathology and immunohistochemistry are useful for establishing a definitive diagnosis. This report describes an extremely rare case of a deep dermal and subcutaneous canine hemangiosarcoma in the perianal area.

## Case presentation

**Fig. 1:**
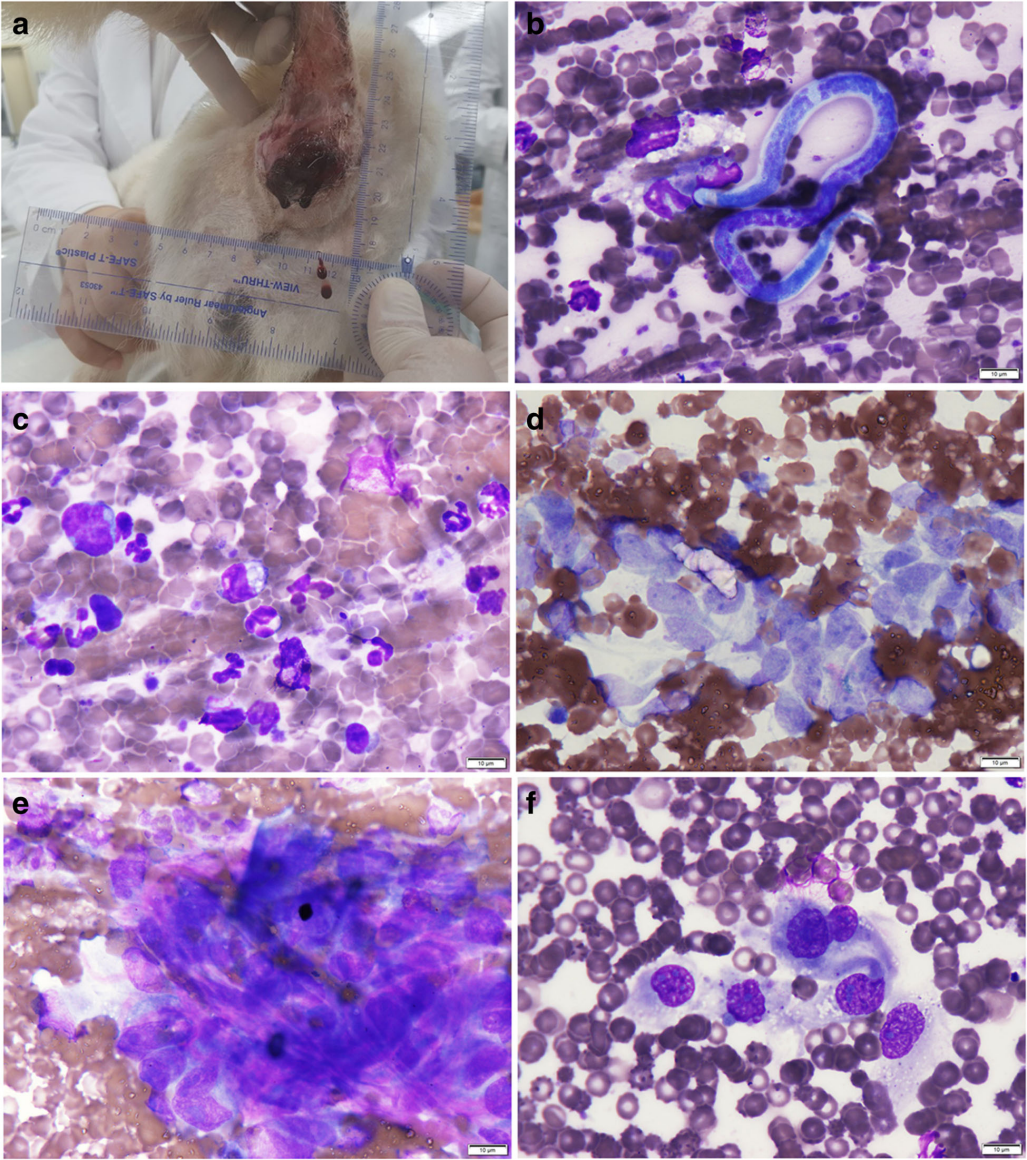
**a** Ulcerated and hemorrhagic mass in the right perianal region. **b**–**f** Fine-needle aspiration of the mass; **b** microfilaria and neutrophils. **c** inflammatory cells. **d** spindle shape of neoplastic cells with multiple nucleoli. **e** extracellular eosinophilic matrix and neoplastic mesenchymal cells with distinct multiple nucleoli. **f** increased N:C ratio, macrokaryosis, anisokaryosis, anisonucleoliosis, and distinct multiple nucleoli (Diff-quick stain, × 1000, scale bar: 10 μm)

A 13-year-old intact male spaniel was presented for the evaluation of a 4 × 4 cm, ulcerated, and hemorrhagic mass, since 3 months, in the right perianal region (Fig. 1a). The mass was flat and hard 2 months ago, but had increased in size since then. Two days before the visit to the hospital, it had ruptured and shown hemorrhage.

A CBC test revealed regenerative, mild microcytic normochromic anemia (RBC: 5.09 M/μl, RI: 5.6–8.8; HCT: 30.7%, RI: 37.3–61.7; MCV: 60.3 fL, RI: 61–73.5; reticulocyte: 1.6%, RI: 0–1.2). A serum biochemistry profile showed increased ALT and GGT concentration (ALT: 193 U/L, RI: 10–130; GGT: 10 U/L, RI: 0–7). In the abdominal radiograph, the presence of fecal stasis in the descending colon was observed, and the presence of feces in the anus could not be verified because of the mass. A fine-needle aspirate of the perianal mass was performed and stained with Diff-quick stain for cytologic evaluation (Fig. 1b–f).

A large number of erythrocytes as hemorrhagic manifestation with many microfilaria were observed throughout the slide. Erythrophagia and inflammatory cells such as neutrophils, eosinophils, monocytes and macrophages were also observed. In some of the fields, cell populations derived from mesenchymal origin with high-grade malignancy were seen (increased nucleus to cytoplasm ratio, macrokaryosis, anisokaryosis, anisonucleoliosis, and distinct multiple nucleoli). Low cellularity, eosinophilic materials outside cells, and cytoplasmic appearance suggested that the cells were derived from mesenchymal origins. Thus, malignant mesenchymal tumor with inflammation and heart worm infection was the diagnosis.

**Fig. 2:**
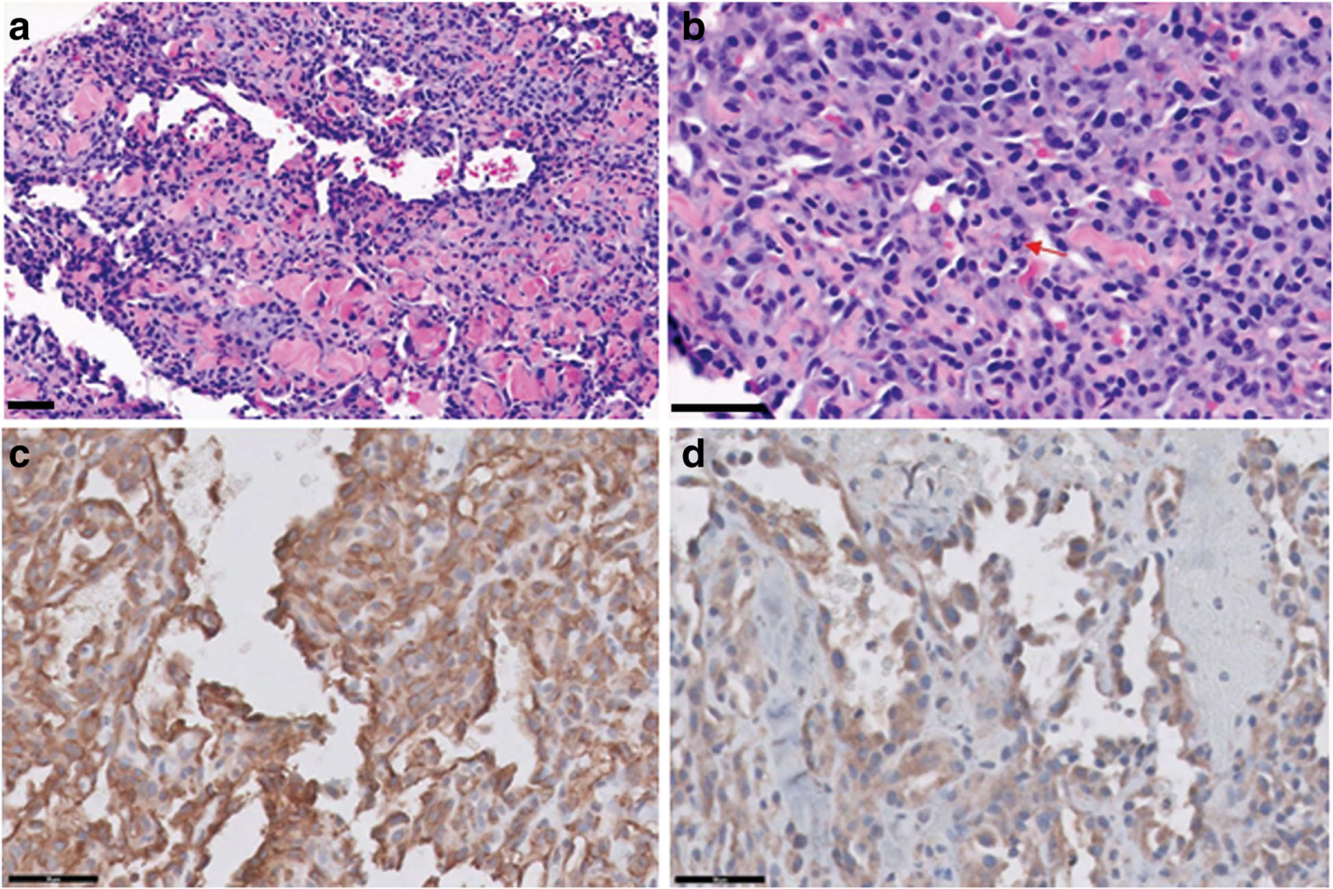
**a** Histologic section of a deep dermal and subcutaneous hemangiosarcoma (H&E, × 200). **b** Mitotic figure (arrow) (H&E, × 400). **c** Immunohistochemistry of a deep dermal and subcutaneous hemangiosarcoma (CD31, × 400). **d** Immunohistochemistry of a deep dermal and subcutaneous hemangiosarcoma (factor VIII-related antigen, × 400). Scale bar: 50 μm, H&E: hematoxylin and eosin

Based on the results of cytologic evaluation, punch biopsy of 3-mm size was conducted at three sites under locoregional anesthesia with lidocaine spray and bupivacaine intralesional injection (< 2 mg/kg) and the biopsy samples were submitted for histopathologic evaluation (IDEXX Laboratories, Inc., Lenexa, KS, USA). Deep dermal and subcutaneous hemangiosarcoma (3 mitotic figures/10 high power field (HPF, 400×)) was diagnosed and histopathological findings were as follows: the specimen was characterized by a poorly demarcated and non-encapsulated proliferation of atypical vascular endothelium (Fig. 2a and b). These cells proliferated as tortuous sinusoids or capillary like structures within the dermal connective tissue. There was an invasion up to the level of the deep dermis and subcutaneous tissue. Individual cells were characterized by scanty amphophilic to eosinophilic cytoplasm and mild to moderate pleomorphic, euchromatic nuclei with variably sized nucleoli. Immunohistochemistry results revealed strong cytoplasmic staining for cluster of differentiation 31 (CD31) and moderately strong cytoplasmic staining for factor VIII-related antigen in the neoplastic cells (Fig. 2c and d).

## Discussion and conclusions

Tumors of the perianal area occur frequently in dogs, and the two most common tumors are perianal gland adenoma and anal sac adenocarcinoma [1]; others such as mast cell tumor, lymphoma and melanoma can also occur at this site.

In the cytological evaluation of the perianal mass from this case, the characteristics of epithelial cell population were not observed. The cellularity of aspirates was low; the spindle shape of neoplastic cells and extracellular eosinophilic matrix indicated that these cells were mesenchymal in origin. Cytologic criteria of malignancy such as increased nucleus to cytoplasm ratio, anisokaryosis, coarse chromatin pattern, multiple nucleoli, and anisonucleoliosis were observed.

Therefore, high-grade malignant mesenchymal tumor was suspected. For definite diagnosis, histopathological examination was performed; biopsy and histopathology can provide a more specific diagnosis as to the origin of tissue [2]. Since reactive fibroblasts may also show many of the cytologic criteria of malignancy with inflammatory cells, a definitive diagnosis of neoplasia based on cytology was not made in the inflammatory lesions [6]. Deep dermal and subcutaneous hemangiosarcoma was diagnosed by histopathological evaluation. Despite no remarkable finding through thoracic radiography, additional examinations such as ultrasonography were not performed because of the owner’s inability to cover cost.

Hemangiosarcoma is a very aggressive malignant tumor of blood vessel cells, with 3 types including dermal, hypodermal, and visceral. The visceral form (splenic or cardiac) is most common; it has been reported that the prevalence of hemangiosarcoma is 0.3–2.0% and primary dermal and hypodermal hemangiosarcoma account for 14% of these tumors in dogs [7]. A retrospective study of 212 dogs with cutaneous hemangioma and hemangiosarcoma reported that hemangiosarcoma had a remarkable predilection for the dermis compared to the subcutaneous region (73 and 7%, respectively) [8]. Dermal hemangiosarcoma commonly occur in the ventral abdomen or prepuce, but hypodermal hemangiosarcoma do not seem to have a site predilection [7]. According to the WHO classification, locally infiltrative lesions with irregular vascular channels are classified as hemangiosarcomas [9]. The histological findings of the perianal mass of this case resonates with the characteristics of a hemangiosarcoma. It was also confirmed by immunohistochemistry results for CD31 and factor VIII-related antigen. CD31 and VIII-related antigen are well-established endothelial cell markers [10]; CD31 is expressed by megakaryocytes, platelets, and endothelial cells, and VIII-related antigen is expressed by endothelial cell Weibel-Palade bodies and megakaryocytes [11]. The protocol of immunohistochemistry for CD31 and VIII-related antigen in samples from dogs and cats are described in detail in previous studies [11, 12].

Immunohistochemistry using antibodies for the detection of specific antigens in the tissue sections is an effective tool to obtain a final diagnosis [13], and immunohistochemical markers such as CD31 and VIII-related antigen enable the confirmed diagnosis of hemangiosarcoma [11].

Deep dermal and subcutaneous hemangiosarcoma in the perianal region is a rare condition, and its prognosis is usually poor. Diagnosis using cytology or histopathological examination is important because the prognosis depends on the type of tumor in the perianal region. Perianal gland adenoma and anal sac adenocarcinoma are the most common tumors in the perianal regions, but other different types of tumors may also occur as in this case; therefore, accurate diagnosis is required using cytology and/or histopathological examination.

## Abbreviations

ALT: Alanine aminotransferase
CBC: Complete blood counts
CD: Cluster of differentiation
GGT: Gamma-glutamyl transferase
H&E: Hematoxylin and eosin
HCT: Hematocrit
HPF: High power field
MCV: Mean corpuscular volume
RBC: Red blood cell
RI: Reference interval
WHO: World Health Organization
